# A Report of a Case of Reactivation of Plaque Morphea After Sunlight Exposure and a Literature Review

**DOI:** 10.7759/cureus.70879

**Published:** 2024-10-05

**Authors:** Zlatina Ivanova

**Affiliations:** 1 Dermatology and Venereology, Medical University of Plovdiv, Plovdiv, BGR

**Keywords:** literature review, morphea, reactivation, sunlight, triggering factor

## Abstract

We present the case of a 61-year-old woman with a histopathologically confirmed diagnosis of plaque morphea. The disease began about four years ago with the appearance of a single oval sclerotic plaque located on the front surface of the right lower leg. After topical treatment, the plaque underwent reverse development. Two years later, after intense exposure to the sun, reactivation of the plaque occurred with the development of a peripheral reddening of the "lilac ring" type, thickening, and induration of the affected skin. After topical treatment, the plaque regressed again after six months. The next summer, the patient regularly used topical photoprotective agents and avoided prolonged exposure to solar radiation, during which no new reactivation of the plaque occurred. The observed case suggests that UV light (sunlight) could be a triggering factor for the reactivation of plaque morphea.

## Introduction

Localized scleroderma (LoS) or morphea is a connective tissue disease that affects a limited area of ​​the skin and subcutaneous tissues with the development of an initial inflammatory reaction, followed by fibrosis and finally atrophy. Five clinical forms are described - plaque, linear, deep, bullous, and generalized. The disease is self-limiting, with spontaneous regression occurring after three to five years. Some patients may develop relapses [1]. According to the study by Mertens J, including 344 patients with LoS, disease recurrence was observed in 27% of patients with early onset in childhood and 17% of patients with late onset in adulthood [2]. Linear LoS recurred most frequently, regardless of age at disease onset. The average time between the onset of the disease and the beginning of the relapse is 26-27 months, and the time until the occurrence of repeated remission is eight to nine months.

## Case presentation

**Figure 1:**
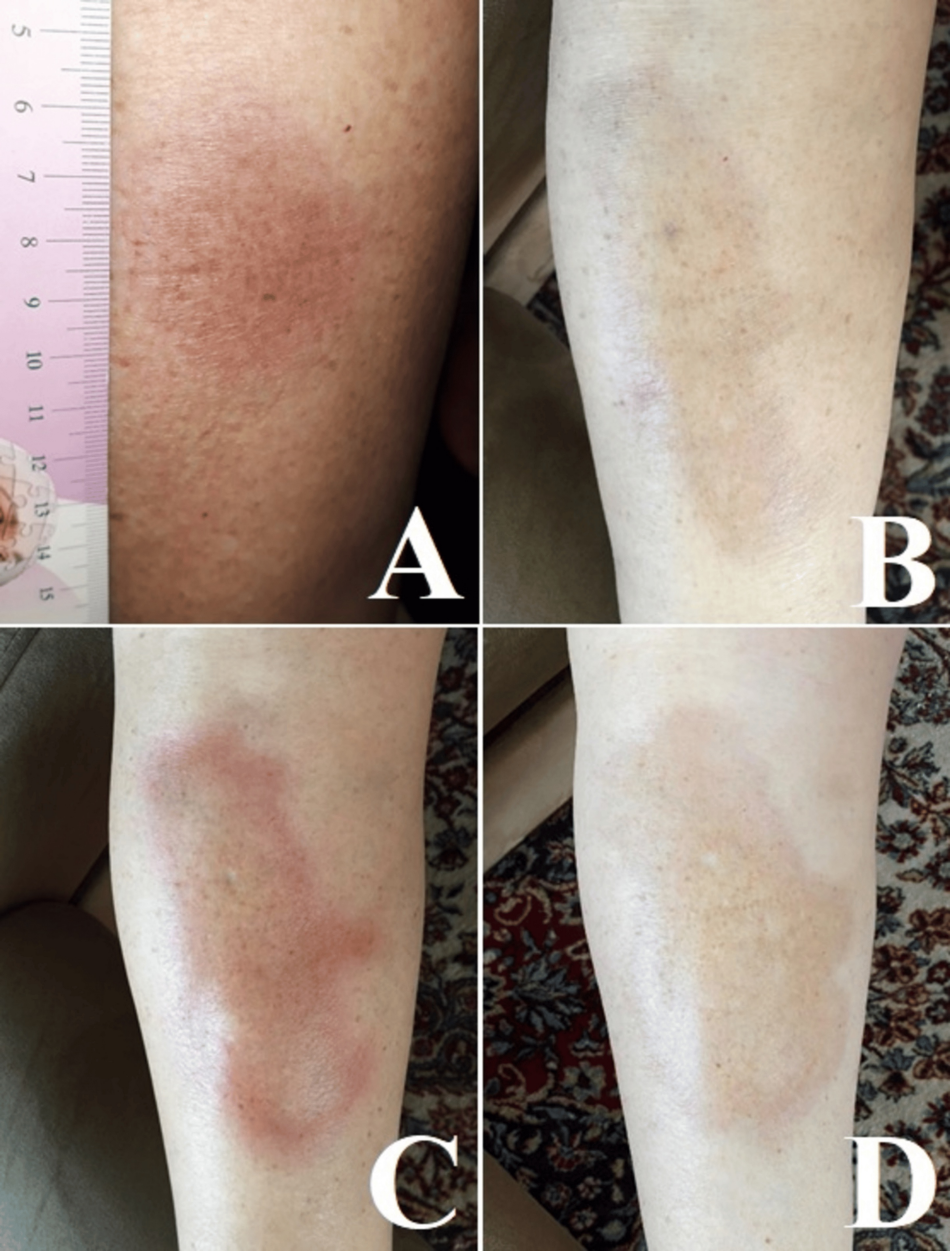
Development of morphea plaque over time (A) Initial morphea lesion on the right leg. (B) Morphea plaque after remission. (C) Morphea plaque after reactivation. (D) Morphea plaque after second remission.

We present the case of a 61-year-old woman who, about four years ago, developed a single, red-brown spot measuring 4x3 cm on the anterior surface of the right lower leg (Figure 1A). Topical treatment with clobetasol propionate 0.05% ointment followed by tacrolimus 0.1% ointment was applied, but over the next six months, the spot gradually enlarged and the skin thickened and hardened. The dermatological examination revealed an elongated plaque 8x4 cm in size, reddish color, and skin induration. Histopathological examination was performed, which showed the presence of hyperkeratosis, epidermal atrophy, a smoothed dermo-epidermal boundary, thickened and homogenized collagen fibers in the dermis, mild perivascular infiltrates, and eccrine sweat glands located among collagen. Laboratory tests, including immunological, are normal. A diagnosis of localized plaque scleroderma (plaque morphea) was made. Phonophoresis treatment with clobetasol propionate 0.05% ointment for two weeks followed by a long-term topical Madecassol 1% cream (phytoproduct containing *Centella asiatica*) resulted in plaque regression after about six months (Figure 1B). In September of the next year, the patient came again for examination due to a change in the skin lesion. During the examination, a slight enlargement of the lesion, peripheral reddening of the "lilac ring" type, skin thickening, and induration were detected (Figure 1C). According to the patient, the changes occurred after intense exposure to the sun during a summer vacation. In addition, the patient reported having had a sun allergy for many years. The previous treatment with clobetasol propionate 0.05% ointment and Madecassol 1% cream was repeated, and remission was observed after about six months. The next summer, the patient applied photoprotection and avoided prolonged sun exposure, during which no new recurrence was observed (Figure 1D).

## Discussion

LoS can have a chronic-relapsing course. There are published studies on relapse rates, but little data exist on the factors that trigger them [1,2].

Reactivation after successful treatment

O'Brien J, et al. followed up 130 adults and children with LoS for eight years [3]. In 29% of patients, relapses were observed, which occurred on average 1.7 years after documented disease inactivity. Relapses were more common in adults and patients with generalized LoS compared to children and patients with linear LoS. In patients treated with methotrexate, relapses occurred later (on average after 2.2 years) and were less frequent (27%) compared to those in patients treated with UV-A1 phototherapy, after 1.4 years and 39%, respectively. Some patients had more than one relapse.

Vasquez R, et al. also reported a high incidence (46%) of LoS recurrence after successful UV-A1 phototherapy [4].

Fan W, et al. observed recurrences of linear LoS of the scalp in 10 (41%) of 24 patients after discontinuation of systemic therapy. In one (16%) of six patients, reactivation occurred after injections with fillers administered to improve the facial contour [5].

Pregnancy-associated reactivation

LoS reactivation was observed in three out of 10 women during their pregnancy [6].

Drug-induced reactivation

Pettit C and Mosser-Goldfarb J reported the case of a 16-year-old girl with a linear LoS located on the back of her left leg several years ago. On the occasion of inflammatory acne, the patient was started on minocycline treatment, and two weeks later, the linear lesion was reactivated. Simultaneously, two new active scleroderma plaques appeared on the chest and abdomen. Reversal of lesions in several months occurred after discontinuation of minocycline and treatment with mometasone 0.1% cream and tacrolimus 0.1% ointment [7].

Alegre-Sánchez A, et al. observed a recurrence of morphea in a 61-year-old woman that occurred two months after initiation of nivolumab immunotherapy for lung adenocarcinoma [8].

Reactivation after treatment procedures

Wang H and Long X reported the case of a man with a nine-year history of LoS on the right cheek that resulted in fibrosis and atrophy of the skin in the affected area. Six months after treatment with autologous fat transplantation, the appearance of new lesions in the forehead area was observed [9].

Reactivation after administration of a vaccine against COVID-19

Gungor M and Bilgic A reported a case of plaque morphea with a 10-year remission that recurred two weeks after vaccination with the mRNA vaccine for COVID-19 with the appearance of new lesions in the areas of the previous lesions [10].

Analysis of reported cases shows that recurrence of LoS may be associated with both exacerbation of old lesions [4-7] and the appearance of new scleroderma lesions [7-9]. In their study, O'Brien et al. [3] observed that 82% of LoS recurrences were at the site of the primary lesions and were manifested by either enlargement of existing lesions or the development of new plaques at the same anatomic site. Measurement with the specific disease assessment scales (LoSCAT disease activity components) showed that relapses were milder than initial activity, perhaps because they were diagnosed earlier.

Our patient reported that two years after the onset of the disease, when remission leading to an atrophic dyschromic lesion was achieved, a reactivation occurred, represented by the development of skin thickness and induration in the affected area and the appearance of a "lilac ring", which she associated with abundant exposure to sunlight radiation. After achieving a repeat remission, the patient regularly used photoprotective agents during summer, and when exposed to the sun, no new relapses were observed.

Our observation suggests that UV light (sunlight) could be a trigger for LoS recurrence. This is contrary to the fact that one of the common treatment methods for LoS is phototherapy with the application of UVA, UVA1, PUVA, and less often narrow-spectrum UVA and UVB rays [11-13]. Phototherapy is used for its immunosuppressive and anti-inflammatory effects. In patients with LoS, it was found that the UV-A/UV-A1 spectrum decreases collagen type 1, collagen type 2, and transforming growth factor-beta (TGF-beta), and increases the production of collagenase (MMP-1) and IFN-gamma [14]. Sunlight can also increase the hyperpigmentation of scleroderma skin because of which the use of sunscreen is recommended. Patient-reported photosensitivity could also be a contributing factor in scleroderma reactivation. According to Vasquez R, et al. the higher Fitzpatrick skin type (III-V) was protective for the risk of recurrent morphea activity [4].

## Conclusions

After achieving LoS remission, relapse may occur. Establishing the trigger factors for the reactivation of the pathological process would help in its prevention and a better prognosis of the disease. The possibility of UV radiation (sunlight) being a triggering factor is a reason to recommend the use of photoprotective agents in patients with morphea.
